# Human Papillomavirus Prevention by Vaccination: A Review Article

**DOI:** 10.7759/cureus.30037

**Published:** 2022-10-07

**Authors:** Samruddhi H Charde, Rupesh A Warbhe

**Affiliations:** 1 Department of Pharmacology, Jawaharlal Nehru Medical College, Datta Meghe Institute of Medical Sciences (DU), Wardha, IND

**Keywords:** awareness, vaccine, detection, cancer, cervical, efficiency, safety

## Abstract

Cervical cancer is the most common cancer among women worldwide. It is caused by infection due to human papillomavirus. There are some screening tests available for its detection. Early detection can increase the chances of survival. The HPV vaccine offers a long-lasting, reliable defence against the HPV infection that is most frequently linked to cancer. HPV 16 and HPV18 are high-risk cancer-causing strains. It is transmitted most commonly through the sexual route. There are horizontal and vertical modes of transmission of the infection present. Vaccines have been introduced to prevent cervical cancer in the market. Awareness and knowledge of vaccines are lacking in people. Vaccine awareness can be made in schools, colleges or among their parents. Social media is vital in spreading knowledge faster in today's generation. The number of precancers of the cervix in young women has been reduced due to HPV vaccination. A practical and safe method to prevent HPV is early vaccination. Vaccines provide cell-mediated immunity. It is more helpful if the vaccine is taken at a younger age. It can only prevent, not treat, cervical cancer. General practitioners are essential in informing people of the vaccine and its potential benefits. There are many ongoing types of research going on in this field so that people can get relevant information. The objective of this review is to provide information about the research till now, knowledge among people, the need for vaccines, effects and safety of vaccination. Research is going around the world to prevent cancer, which can decrease the worldwide burden of deaths due to cancer. Among all types of cancer, vaccines are available only for preventing cervical cancer, which has a high prevention rate.

## Introduction and background

The second most common diseases worldwide that lead to death are related to cancer. Human papillomavirus (HPV) is a DNA virus, a nonenveloped double-stranded one that belongs to the family Papillomaviridae [1]. HPV is an infectious agent mainly transferred sexually. In both men and women, the virus may cause cancer associated with HPV. HPV infection can be associated with neck and head cancer, apart from cervical cancers. There has been significant improvement in the prevention and testing of cervical cancer in the last 10 years [2].

Vaccines are available for HPV to prevent cervical cancer. Some benefits are the potential effects of HPV vaccination. World's 5% of cancers are associated with HPV. Cervical cancer is considered a significant worldwide load due to HPV. Vaccination is essential to reduce the deaths occurring due to HPV worldwide [3]. Transfer of HPV takes place by skin-to-mucosa or skin-to-skin contact. Transference through sexual contact is observed in most cases; other modes of transmission include fingers, fomite, mouth, and skin contact. Transfer from mother to children is in the form of vertical transfer of HPV. Transmission of HPV through water is not seen [1]. HPV infection is more likely to occur in teenage women and 20-30 years. Its chances increases if sexual intercourse is started at an early age or with different sexual partners [4].

Innovation and research concerning detecting and treating cancer can reduce the burden of healthcare all over the world. A vaccine named Gardasil is available in the market. The risk of women dying from cervix cancer will decrease if it is caught in the initial stage [4]. Based on some research, we get to know that the use of the vaccine is beneficial, effective, and produces protection [5]. Whereas according to some articles efficacy of the vaccine is under process. Trials are taking place to see the potency of the vaccine that has been found till now [6].

The HPV vaccine is safe and successful in preventing cancer of the cervix in the female. Apart from this, the infection can also cause oral cavity, penis, and anus cancer, but studies are lacking in assessing the safety and efficacy of the vaccine in males [7]. Telling people the benefits of vaccines based on evidence that they are safe to use depending on previous studies makes them able to rely on their use. A wide range of individuals is responsible for providing information to the public, such as the media, healthcare people, teachers, and stakeholders, so they should be given correct information about the risk and beneficial effects of the vaccine [8].

According to studies comparing the mono-, bi-, and quadrivalent preventive HPV vaccines, women who receive the shots between the ages of 24 and 45 had lower levels of neutralising antibodies. As a result, young women between the ages of 15 and 26 make up the leading target group for HPV vaccination. Therefore, it is advised to take the vaccine at the ages between 15 and 26. But it is not advised to take vaccines by pregnant women. HPV vaccine stimulates the body to produce antibodies that, in future encounters with HPV, bind to the virus and prevent it from infecting cells. Approximately 70% to 90% of all cancer related to HPV can be prevented by vaccination. Women of 25 years to 65 years old are advised for screening. Gardasil 9 nonvalent vaccine, Cevarix bivalent vaccine, and the Gardasil quadrivalent vaccine are available in the market. The vaccine Gardasil is available on market and is to be taken in three doses, which need not be repeated in life. The recommended dose is two months between doses 1 and 2, with a minimum of four weeks; six months between doses 1 and 3, with a minimum of 24 weeks; and a minimum of 12 weeks between doses 2 and 3.

## Review

Human papillomavirus

HPV causes infection in the genital region of women and men. Some viruses can also cause disease in the throat and mouth lining. Incidences of cancer of the cervix have a relation to the age group. The maximum cases are seen in the age group of 50 to 54. Also, the incidences of dying due to cancer increase as the age increases; this shows that in the young age group people, the progression of infection to cancer is slow [9].

Cervical cancer is a serious health problem in developing and developed countries [10]. When the HPV virus remains in the skin, the genital wart can develop. Small growths or bumps on the genitals are genital warts. The development of cervical cancer increases with the occurrence of genital warts [11].

In comparison to females, the cause of HPV in males is different. Ethnicity, race, not using a condom, multiple sex partners, other sexual behaviours, and human immunodeficiency (HIV)are the most common factors related to the infection of HPV in men. It is said that clearance of HPV is lower in uncircumcised men than in circumcised men [11].

In their lifetime, sexually active women and men will get HPV infection; approximately 75% of those. Therefore, it is said to be commonly caused by sexual transmission. As sexual risk attitude is more in the younger adult age group, infection is likely to occur in the younger adult age group. Sexually active women have a high risk of carrying the condition. Various types of HPV strains are high-risk types linked to cancers and some low-risk types cause benign diseases. HPV-6 and HPV-11 are responsible for about 90% of genital warts, and HPV types 16 and 18 cause approximately 70% of cervical cancer [12].

Detection of infection

Cervical cancer rates are decreased by 50% to 80% due to the proper organising of screening programmes at the population level. The main reason for dramatic mortality and incidence of cervical cancer is unequal access to screening programmes. The screening for cervical cancer is by identifying women with no symptoms with precancerous lesions and then getting a chance to diagnose and treat cancer before it develops. Cervical cytology (Pap testing), co-testing, HPV primary screening using HPV, and cytology lower cervical cancer mortality and incidence if procedures are obeyed. Furthermore, there are some disadvantages and advantages of the different screening tests. Screening is advised for women of 25 years to 65 years old [13].

Since the reason was identified, cervical cancer screening has been made easier. In addition to cytology-based tests, HPV-based testing is a crucial component of cervical cancer screening [14]. Dying due to cervical cancer can decrease if women do screening for cervical dysplasia (CD) and HPV. The screening will be more effective if low-cost, rapid and new screening techniques are used. Digital colonoscopy of high resolution can be done with HPV screening to detect CD [4].

People’s attitude and behaviour towards vaccination

The use of the HPV vaccine is lagging behind than that of the other vaccines available on the market for teenage group people. There are different reasons for not taking the vaccine. Various myths are prevailing in society, and misconceptions about the vaccine are there in the minds of people. Some believe that the immune system naturally clears the infection. To make clear the myths prevailing in society, research on this topic is essential, scientifically proving the safety and effectiveness of the HPV vaccine [15]. The introduction of the vaccine of HPV took place more than 10 years ago. Some researchers have provided about how a provider can address the question effectively and make the concerns of parents clear about the use of the vaccine. Messages that were research tested were given to the providers to address the problem of the parents about the HPV vaccine [16]. For the analysis of the knowledge present in society about accepting the HPV vaccine amongst the unvaccinated and vaccinated, some research has been done and is needed for people who are unaware of it. And in that research, they saw that the unvaccinated adolescents had gaps in the knowledge of the HPV vaccine compared to those vaccinated [17].

People's attitude towards health and daily lifestyle, like alcohol consumption, cigarette smoking, and lengthy exposure to harmful radiation, increases the risk of causing cancer. People are unaware that most cancers occurring worldwide are due to parasites, bacteria, and viruses. The most typical infection transmitted worldwide sexually is HPV [3]. HPV is responsible for 99% of cervical cancers approximately. The transmission can occur through different routes horizontally, vertically, or in self-inoculation. Vaccination of HPV is a measure taken where investment will give a high return [1]. Roughly calculating the effect and safety of the vaccine was the objective of some of the studies. The protocol does not show any risk to the subject, but still, there are limitations in the research [6]. So, the objective is to make people aware of the vaccine and prevent HPV infection in the community.

A research study was conducted among postgraduate residents and dental students to see the acceptance, knowledge, and awareness of the HPV vaccine. It was found that there is a lack of understanding and knowledge. It took place during the curricular module devoted to immunisation practices. A significant population of groups agreed vaccines are effective, but only a few decided they were safe. According to previous studies, they were not interested in discussing or recommending the HPV vaccine [18].

Some reported that lack of information and negative perception about the efficiency and safety of vaccines was in the mind of consumers and their parents, which refuses them from taking vaccines. Some parents think their sons and daughters are not at risk of infection, and there is no need for vaccination. The general practitioner's role is to inform people about it and recommend them to take the vaccine [10].

There was a positive attitude in some students, generally regarding HPV vaccine vaccination, but information about the HPV vaccine, HPV infection and cervical cancer in their community was insufficient. The high vaccine cost was one of the barriers to taking the HPV vaccine [19].

In one study in India, it was seen that doctors do not feel comfortable talking about a girl's reproductive life with the parents of adolescents. Apart from this, they believe that the parents will ignore vaccine recommendations [20]. There is increased awareness about the HPV vaccine HPV due to increasing attention on social media platforms. We can use social media to increase the knowledge among the population about cervical cancer and vaccination, which will help to reduce the reluctance to take the vaccine and encourage vaccine and decrease the burden of cancer occurrences [21].

**Figure 1:**
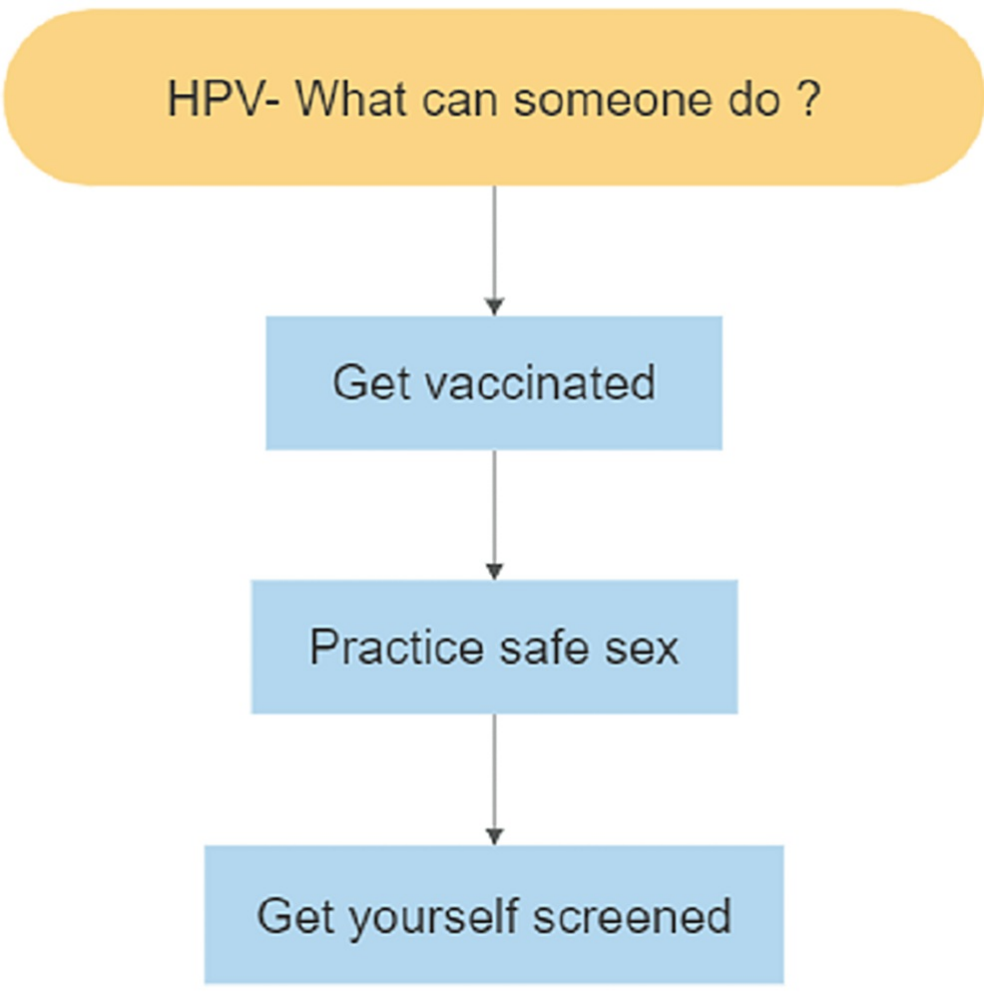
How to prevent human papillomavirus

People have misconceptions about advantages and disadvantages, and knowledge gaps are seen. Concerns about convenience, access and cost were some of the barriers [22]. If a person gets vaccinated, gets himself screened and practices safe sex, then HPV can be prevented (Figure 1).

Need of vaccination

Vaccination is a safe and efficient way to prevent HPV infection and related malignancies. Future studies should concentrate on creating specialised strategies to boost HPV vaccination rates among guys across all racial/ethnic groups. Male teenagers who are members of racial and ethnic minorities are more likely to begin vaccination even though advice is generally the same across these groups [23].

Communicating about the effectiveness of vaccines and cancer prevention is required to tackle people's concerns about vaccination [16]. It motivates the parent to get their child vaccinated. Approximately 70% to 90% of all cancer related to HPV can be prevented by vaccination. The vaccine cannot treat the infection caused by a virus; it can only avoid the infection in an individual. The vaccines have a different effect depending on the type of strain of HPV as a target [3]. Some vaccines have come into existence to stop the spreading of HPV and other related diseases, such as the Gardasil 9 nonvalent vaccine, the Cevarix bivalent vaccine, and the Gardasil quadrivalent vaccine [24].

In June 2015, the European Union authorised the marketing of the Gardasil 9 vaccine in the European medicines agency. Adolescent immunization is the public health target to provide maximum protection from HIV and related diseases [25].

The most common and high-risk types of HPV are HPV16 and HPV18. The use of the vaccine can prevent them. As people enter adulthood, they should be educated by healthcare professionals about vaccination. They will subsequently be educating more. As there is a knowledge gap amongst that age group people [26].

In 2015, an international independent multidisciplinary board, The Human Papillomavirus (HPV) Prevention and Control (HPV-PCB), was created. A team of specialists known as the HPV-PCB offers evidence-based advice on strategic, technical, and policy challenges that arise throughout the execution of HPV reduction programmes. The HPV-PCB seeks to produce and spread a wide range of people with pertinent information on the stakeholders' prevention and management of HPV-associated disorders. By holding two meetings a year, it meets its goals [27].

Safety of vaccine

The occurrence of any adverse effect after taking the vaccine in the participants is a characteristic of the safety of the vaccine. Congenital anomalies, significant disability, long-time hospitalisation, life-threatening experience or death are events termed under severe adverse effects [24].

Prenatal vaccinations are crucial for both the mother's active immunity against major infectious diseases and the neonate's passive immunity against contagious diseases with high morbidity and mortality rates. Live vaccinations are often not advised during pregnancy since they could result in foetal viremia or bacteremia. Most inactivated vaccinations are secure [28].

In a study, they summarised the most recent information on HPV vaccinations created at the start of the twenty-first century. The immunizations have no adverse side effects and are highly safe and effective. In Sweden, both boys and girls were vaccinated as a preventive measure in schools. They provided two doses of the nonvalent vaccine to this age group. The main goal of the immunization was to lower the incidence of genital cancer and the lesions that serve as its precursors. The cervix, which is the site of the most significant malignancy linked to HPV, has primarily been researched. Genital warts are problematic benign ailment that is difficult to treat and frequently has psychosexual repercussions [29].

Male HPV vaccination uptake can still be much improved, and more research is required to understand the causes of racial/ethnic variations in the factors that make vaccination easier. Given that HPV vaccination for men is still relatively new, there is a great chance to advance vaccine developments and public policy, such as teaching in schools and using social marketing to reach male teenagers [23]. Additionally, the majority (97.1%) thought the HPV vaccine was safe, with an overall mean score of 8.8 on a scale from 1 to 10 in a study [10]. During the analysis of the vaccine, no participant died [30]. 

Effect of vaccine

The HPV vaccine was frequently or consistently recommended by the vast majority (81.5%) of GPs who assisted girls between the ages of 11 and 12; this behaviour was more likely to occur in those who thought the vaccine was very effective in preventing HPV-related diseases in girls between the ages of 12 and 26. If GPs frequently or constantly recommended the HPV vaccine to girls aged 11-12, thought the vaccine was highly efficient at preventing HPV-related diseases in boys aged 12-26 and thought the HPV vaccine was very safe, they were more likely to do the same for boys aged 11-12. GPs should be informed about the HPV vaccine to communicate with their patient group and increase coverage rates regularly [10].

Infections with HPV, cervical cancer, precancerous cervical lesions, and genital warts have declined in Sweden [29]. The most significant decreases in disease incidence within the first 25 years of the introduction of HPV4 are believed to result from the prevention of HPV 6/11-related genital warts because cervical malignancies have a longer natural history [31].

The concern is raised that differences in vaccination completion could increase already-existing disparities in cervical cancer due to black patients' lower completion rates compared to white patients. The vaccine is indicated in a teenage group of the population. The vaccine ensures maximum protection if all the doses are taken [32].

Regarding the participants' attitudes, girls aged 12-26 years had a mean score of 9.2 and boys had a mean score of 8.1 for the effectiveness of the HPV vaccine in preventing related diseases. Almost two-thirds (59.9%) and one-third (32.6%) of the participants had higher scores on a 10-point Likert-type [10].

The quadrivalent and bivalent HPV vaccinations offer high levels of protection against enduring HPV16 and HPV18 infection. According to studies comparing the mono-, bi-, and quadrivalent preventive HPV vaccines, women who receive the shots between the ages of 24 and 45 had lower levels of neutralising antibodies. As a result, young women between the ages of 15 and 26 make up the leading target group for HPV vaccinations. Additionally, earlier studies demonstrated that Cervarix, a preventative vaccine, offered more excellent protection against HPV infection than Gardasil. Compared to Cervarix, the levels of anti-HPV16 and anti-HPV18 antibodies were much lower after using Gardasil. These findings imply that a booster dose of the vaccination and the addition of appropriate adjuvants can enhance immune responses and raise levels of neutralising antibodies [11].

According to a study, introducing the HPV vaccine on a big scale is doable, and the vaccine is trusted and safe by the public [33]. According to an analysis, none of these reviews performed meta-analyses; instead, they qualitatively summarised their findings [34].

This accomplishment is viewed as a steppingstone in lowering the prevalence of cancer linked to HPV. The impact of vaccination campaigns is determined by the coverage levels attained, implementation techniques (with or without catch-up), and recommendations for vaccine use. Therefore, comparing pre-and post-vaccination HPV prevalence is crucial to assess the vaccine's efficiency and its effects on our community. This comparison should consider changes in women's sexual behaviour because the HPV transmission route is sexual [35].

According to our model-based findings, a catch-up vaccination programme that included males and females aged 9 to 45 would promote public health and be cost-effective for men and women up to age 34 and for women up to age 45. Vaccination following the most recent ACIP recommendations, which include catch-up vaccinations through age 26, may be considered almost cost-effective. Mid-adult vaccination unquestionably has value for some populations, given the variety of risk and the susceptibility of mid-adults to HPV infection, which develops into a disease that may have been averted with the vaccine. Our findings back up shared clinical decision-making through age 45 and catch-up immunisation to age 34 [36].

Research demonstrates the possible financial benefits of 9vHPV immunisation in Spain. Comparing the deployment of the 9vHPV vaccination programme to the current 4vHPV vaccination programme reveals that the 9vHPV vaccination programme is more affordable and can offer significant additional public health advantages [37].

## Conclusions

This review showed us that some types of HPV can cause cervical cancer. It can be transferred easily from person to person through sexual contact if precaution is not taken. One of the methods to prevent this is taking vaccination for HPV. Taking vaccines is an effective and safe method. But the vaccine is contraindicated in pregnancy. Although there are some barriers to vaccine uptake, further studies will probably solve them in the coming years. But according to the studies to date vaccine is said to be providing beneficial effects. A vaccine, which is introduced in the market, is proven to be safe and effective to date for the prevention of cervical cancer in women. The conclusion is consistent with the evidence presented. This review is a source for students to collect knowledge about the prevention of cervical cancer through HPV vaccination. The article provides a description of how useful these practices are and what are misconceptions about this subject.
